# A Proposed Taxonomy of Anaerobic Fungi (Class *Neocallimastigomycetes*) Suitable for Large-Scale Sequence-Based Community Structure Analysis

**DOI:** 10.1371/journal.pone.0036866

**Published:** 2012-05-16

**Authors:** Sandra Kittelmann, Graham E. Naylor, John P. Koolaard, Peter H. Janssen

**Affiliations:** AgResearch Ltd, Grasslands Research Centre, Palmerston North, New Zealand; University of Minnesota, United States of America

## Abstract

Anaerobic fungi are key players in the breakdown of fibrous plant material in the rumen, but not much is known about the composition and stability of fungal communities in ruminants. We analyzed anaerobic fungi in 53 rumen samples from farmed sheep (4 different flocks), cattle, and deer feeding on a variety of diets. Denaturing gradient gel electrophoresis fingerprinting of the internal transcribed spacer 1 (ITS1) region of the *rrn* operon revealed a high diversity of anaerobic fungal phylotypes across all samples. Clone libraries of the ITS1 region were constructed from DNA from 11 rumen samples that had distinctly different fungal communities. A total of 417 new sequences were generated to expand the number and diversity of ITS1 sequences available. Major phylogenetic groups of anaerobic fungi in New Zealand ruminants belonged to the genera *Piromyces*, *Neocallimastix*, *Caecomyces* and *Orpinomyces*. In addition, sequences forming four novel clades were obtained, which may represent so far undetected genera or species of anaerobic fungi. We propose a revised phylogeny and pragmatic taxonomy for anaerobic fungi, which was tested and proved suitable for analysis of datasets stemming from high-throughput next-generation sequencing methods. Comparing our revised taxonomy to the taxonomic assignment of sequences deposited in the GenBank database, we believe that >29% of ITS1 sequences derived from anaerobic fungal isolates or clones are misnamed at the genus level.

## Introduction

Strictly anaerobic fungi of the class *Neocallimastigomycetes* play a pivotal role in the rumen by physically and enzymatically attacking the fibrous plant material ingested by the ruminant animal [1], [2]. By breaking down plant cell wall carbohydrates, such as cellulose and hemi-cellulose, anaerobic fungi deliver readily accessible nutrients, mainly acetate, propionate, and butyrate, to their ruminant host, and large amounts of reducing equivalents in the form of hydrogen (H_2_) to the bacterial and archaeal communities [3], [4], [5]. The initial attack by fungi on plant fiber appears to facilitate a more rapid breakdown of forage feed by fibrolytic bacteria [6], [7]. Anaerobic fungi may therefore be very important for feed utilization efficiency and animal growth of pasture-fed ruminants [8], [9]. However, the H_2_ released by the anaerobic fungi stimulates the activity of methanogenic archaea [10], which convert H_2_ and carbon dioxide (CO_2_) to methane (CH_4_), a greenhouse gas considered 25 times as potent as CO_2_ [11]. Globally, the livestock sector accounts for 18% of total anthropogenic greenhouse gas emissions [12]. In New Zealand, a country with a significant pastoral sector, ruminant-derived CH_4_ alone makes up 32% of the country’s total anthropogenic greenhouse gas emissions [13].

To date, six genera of anaerobic fungi have been described, mainly on the basis of their morphological characteristics: *Anaeromyces*, *Caecomyces*, *Cyllamyces, Neocallimastix, Orpinomyces,* and *Piromyces*. However, the roles of different anaerobic fungi in ruminal CH_4_ formation still remain to be elucidated, as does the definition of their niches. Anaerobic fungi, through their penetration and degradation of plant tissue and their production of H_2_, may actively shape the remainder of the microbial community, such as bacteria, archaea, and ciliate protozoa. By influencing the structures of these communities, and the fermentation pathways they employ, fungi may increase or decrease CH_4_ emission by the host.

In the absence of readily-manipulated models, statistically significant correlations between host phenotype and microbial (including fungal) community structure require analysis of large-scale animal trials. Since microscopic and microbiological methods are tedious and may not in all cases be comprehensive enough to reflect the true diversity and detect subtle shifts in the fungal community *in situ*, molecular monitoring tools are more appropriate for assessing the structure of anaerobic fungal communities [14]. Although scientific interest in the roles of eukaryotes in intestinal environments is rapidly increasing, and sequence information is accumulating, limited attempts have been made to taxonomically assign anaerobic fungi based on sequence information in a systematic way. This aim may in the future be achieved by including sequence data from genes other than the rRNA locus [15]. However, a recent study shows that it may be difficult to find additional regions suitable as markers for anaerobic fungi [16], and until more genome data are gathered, the rRNA locus remains the genomic area for which most sequence information is available.

Fungal small-subunit rRNA genes are not suitable for phylogenetic distinction between the different genera and species of anaerobic fungi, due to their high degree of sequence conservation [17], [18]. The polymorphic and homoplasious internal transcribed spacer 1 (ITS1) region is of limited evolutionary use. It is, however, widely accepted as a molecular marker for the anaerobic fungi in general [17], [19], [20] and proved highly useful for community structure comparisons, e.g., [21], [22]. ITS1 amplicons have been used for fingerprinting analyses such as denaturing gradient gel electrophoresis (DGGE; [22]), an automated method of intergenic spacer analysis (ARISA; [23], [24]), restriction fragment length polymorphism analysis (RFLP; [14]), and size-based selection using Spreadex [22]. Tuckwell *et al.* described sequence motifs of the four variable regions of the ITS1 for all six genera of anaerobic fungi known to date [25]. This method is highly valuable for the placement of new isolates belonging to known genera, but it is not readily applied to large datasets that are not based on sequence alignments. In order to assign large amounts of sequence data from high-throughput next-generation sequencing techniques, a phylogeny-derived and thoroughly curated anaerobic fungal database is desirable that allows for reliable BLAST-based community structure analysis. In a recent study, Fliegerova *et al.* established a rumen fungal phylogeny based on the six known genera, and pointed out that several sequences deposited in the NCBI (National Center for Biotechnology Information) database are mis-named [26]. While these authors chose to assign all ITS1 sequences they retrieved from cow manure to known genera, we believe that the diversity of anaerobic fungi is not yet fully understood. Nicholson *et al.* [22] used DGGE fingerprinting and subsequent sequence analysis of excised bands, while Liggenstoffer *et al.* [21] employed large-scale next-generation-sequencing of fungal ITS1 genes to examine feces of a wide range of wild and domesticated ruminant and non-ruminant herbivores. Both teams discovered several novel clades of anaerobic fungi that may represent new genera and species.

To compare communities using large amounts of next-generation sequencing data from taxonomic marker genes, it is useful if all sequences can be assigned to a common taxonomic rank, for example a level equivalent to species. It does not actually matter if the groups are not exactly biological species, although it would be preferable if they were. What is important is that large undifferentiated groups at higher taxonomic ranks are not created, which is commonly found in schemes that include poorly differentiated sequences in grab-bag categories often labeled as “Other”. That means that an effort needs to be made to differentiate these groups using the albeit limited information that is available. Because microbial taxonomies are incomplete due to the lack of formal descriptions of groups represented only by gene sequence data, interim working taxonomies are required to allow classification of sequences obtained in marker gene surveys. Efforts have been made to achieve such a working taxonomy for bacterial and archaeal 16S rRNA genes [27], and it is widely acknowledged that a similar taxonomic guide is needed to characterize anaerobic fungi.

Here, we constructed ITS1 clone libraries from 11 rumen samples selected based on their distinctive DGGE patterns, and used the 401 clone sequences obtained together with 197 sequences from isolates (including six so far unpublished sequences from three New Zealand isolates), 183 environmental sequences selected from earlier studies [21], [22], [26], [28], and 16 sequences from excised DGGE bands to build an improved taxonomic framework for anaerobic fungi. Due to the limitations of the marker gene, this detailed framework does not try to accommodate all sequences in known genera and species, but instead differentiates novel groups of sequences as groups at approximately species level and applies clustering on genus level only where morphologically described isolates are available. The advantage of this system is that it allows finer-scale changes in anaerobic fungal community structures to be detected. It is an evolving tool, and is not intended to be a definitive statement on phylogeny and taxonomy of the class *Neocallimastigomycetes*. The taxonomy and sequence files produced may be used in future studies for reliable BLAST-based evaluation of data generated by large-scale next-generation sequencing techniques.

## Materials and Methods

### Collection of Samples from Ruminant Animals

The use of animals, including welfare, husbandry, and experimental procedures, and collection of the rumen samples used for this study, was approved by the AgResearch Grasslands Animal Ethics Committee and the Massey University Animal Ethics Committee, and complied with the institutional Codes of Ethical Conduct for the Use of Animals in Research, Testing and Teaching, as prescribed in the Animal Welfare Act of 1999 and its amendments. Rumen samples were collected as part of a series of feeding trials conducted at different institutions in New Zealand under permit numbers 06/119 and 06/126 (Massey University, Palmerston North), and 11110/modification 775 (AgResearch, Grasslands Research Centre, Palmerston North). The animals were kept at AgResearch’s Grasslands Research Centre in Palmerston North, at Massey University, Palmerston North, and at Riverside Dryland Farm, near Masterton.

Samples of whole rumen contents consisting of fluid and solids (approximately 200 g) were collected via rumen fistulae from 4 wether sheep (*Ovis aries*; Romney; flock 1; animals S1 to S4), 5 mature non-lactating dairy cows (*Bos taurus*; Friesian-Jersey cross; animals C1 to C5), and 4 mature castrated red deer (*Cervus elaphus*; animals D1 to D4). These groups were fed with pasture consisting predominantly of perennial rye grass (*Lolium perenne*) and white clover (*Trifolium repens*) during the winter and summer periods. These pasture-fed animals were on that diet throughout the whole season (winter or summer), and one sample was taken per animal per season. The same animals were fed lucerne (*Medicago sativa*) silage (Chaffhage, The Great Hage Company, Reporoa, New Zealand) in a different period during the winter. The animals were adapted to silage for 15 days prior to sample collection and fed twice daily, at 08∶00 hours and 16∶00 hours at a rate of 1.2 times their estimated energy requirements for maintenance.

Rumen samples were also collected from different flocks of sheep fed with a concentrate-based diet (4 Romney wethers; flock 2; animals S5 to S8; [29]), perennial rye grass/white clover pasture during the autumn period (5 Suffolk-Romney cross ewe hoggets; flock 3; animals S9 to S13; [30]), and willow (*Salix* spp.; 5 Suffolk-Romney cross ewe hoggets; flock 4; animals S14 to S18; [30]). All animals had unlimited access to water at all times. The samples were immediately frozen at −80°C and subsequently freeze-dried. The freeze-dried samples were ground in a 100 W household coffee grinder (Russell Hobbs, Mordialloc, Vic., Australia) and stored at −80°C until DNA was extracted.

### Extraction of Nucleic Acids

Nucleic acids were extracted from 50 mg of freeze-dried rumen samples as described earlier [31]. Briefly, cells were disrupted by combined bead-beating (FastPrep FP120; Qbiogene, Carlsbad, CA, USA; 45 s at 6.5 m s^–1^) and phenol-chloroform-isoamyl alcohol (25∶24:1; vol:vol:vol) treatment and subsequent precipitation of proteins with chloroform-isoamyl alcohol (24∶1; vol:vol). DNA was precipitated with 10% (wt/vol) polyethylene glycol-6000, washed with 70% (vol/vol) ice-cold ethanol, eluted in molecular biology grade water and stored at –20°C.

### Primer Design and Validation

The published and newly designed primers used in this study (Table 1) were checked *in silico* for sequence identity using ARB (http://www.arb-home.de; version 5.2, updated September 2010; [32]) with all available 18S rRNA gene and internal transcribed spacer sequence data of anaerobic fungi isolated to date. All primer pairs were validated for specificity by the construction of clone libraries (*n ≥*44).

**Table 1:** Primer pairs used for qualitative and quantitative assessment of anaerobic fungi in New Zealand ruminants.

Use	No. of clones checked to verify specificity	Primer names	Primer sequence (5′ to 3′)	Specificity	Binding position^a^	Reference
DGGE	45	ITS1F	TCCGTAGGTGAACCTGCGG	eukaryote	26 to 44	[49]
		ITS400R^b^	ATTGTCAAAAGTTGTTTTTAAATTAT	anaerobic fungi	373 to 400	This study
Clone libraries	48	ITS1F	TCCGTAGGTGAACCTGCGG	eukaryote	26 to 44	[49]
		ITS400Rw	ATTGTCAAAAGTTGTTTTTAWATTAT	anaerobic fungi	373 to 400	This study
qPCR	44	AF1482F	GAGGAAGTAAAAGTCGTAACAAGGTTTC	eukaryote	–1 to 27	[48]
		AF100R	CAAATTCACAAAGGGTAGGATGATT	anaerobic fungi	100 to 127	[48]
Pyrosequencing	N.D.^c^	MN100F^d^	TCCTACCCTTTGTGAATTTG	anaerobic fungi	105 to 127	[25]
		MNGM2R^e^	CTGCGTTCTTCATCGTTGCG	fungi	416 to 435	[25]

a Numbering according to Tuckwell *et al*. [25].

b This primer was tagged with a 40 bp long GC-rich sequence segment (CGCCCGCCGCGCGCGGCGGGCGGGGCGGGGGCACGGGGGG). at the 5′-end when products were to be separated using DGGE, and the primer was then designated ITS400R-GC.

c N.D., not done.

d Adaptor A followed by a sample-specific barcode was added at the 5′ end of this primer (see Materials and Methods).

e Adaptor B was added at the 5′ end of this primer (see Materials and Methods).

### Assessment of Anaerobic Fungi and Bacteria by Quantitative Real-time PCR

Abundances of total anaerobic fungi in rumen samples were quantified using a Rotor-Gene 6000 real-time rotary analyzer (Corbett Life Science, Concorde, NSW, Australia) and amplicon detection by SybrGreen I fluorescence (LightCycler FastStart DNA Master SYBR Green I Kit, Roche, Auckland, New Zealand). Primers for quantitative real-time PCR (qPCR) amplification are listed in Table 1. Three different plasmids containing the 18S rRNA to 5.8S rRNA gene region inserts of New Zealand isolates *Caecomyces* sp. NZB7, *Piromyces communis* NZB19 and *Neocallimastix frontalis* PNK2 were generated with primers GM1F (5′-TGTACACACCGCCCGTC-3′) and GM2Rm (modified from Li & Heath [20]; 5′-CTGCGTTCTTCATCGTT-3′), combined in equal quantities, quantified with the Qubit dsDNA BR assay kit and fluorometer (Invitrogen, Carlsbad, CA, USA), and used as standards.

Reactions were set up in a Gene-Disc 100 (Corbett Life Science) and sealed with permanent adhesive film (Corbett Life Science). Each template DNA was measured at 4 different dilutions (1∶75, 1∶100, 1∶250 and 1∶500). Since there was no deviation from linearity after correction for the dilution, inhibition of the PCR due to co-extracted materials could be ruled out. Each reaction contained, in a total volume of 20 µl, 2 µl of Light Cycler Mix (Roche), 1 µM of each primer, 2.5 mM MgCl_2_, 4 µg bovine serum albumin (BSA; Invitrogen), and 2 µl of standard or template DNA. The thermal protocol for qPCR amplification and detection was 10 min of initial denaturation (94°C) followed by 50 amplification cycles (30 s at 94°C; 5 s at 60°C; 10 s at 72°C). After each run, melting curves between 72 and 95°C were evaluated for products to assess target-specific amplification. Amplification of bacterial 16S rRNA genes from rumen samples was performed as described earlier [31].

### Amplification of Anaerobic Fungal ITS1 Genes for DGGE Fingerprinting and Clone Libraries

PCR amplification of ITS1 genes from ruminant-derived DNA samples for DGGE and the construction of clone libraries were carried out with primer sets listed in Table 1. Each 50-µl PCR contained 1 × Taq buffer (Roche), 1.5 mM MgCl_2_, 0.75 U Taq DNA polymerase (Roche), 50 µM of each deoxynucleoside triphosphate, 10 µg BSA, 0.5 µM of each primer, and 1 µl of template DNA (10-fold diluted). Non-specific primer binding was minimized with a semi-hot start by transferring the reactions already containing the polymerase from 4°C straight into the pre-heated thermal cycler (94°C). The amplification was performed as follows: initial denaturation at 94°C for 2 min, 35 cycles of denaturing (94°C, 30 s), annealing (52°C, 30 s) and elongation (72°C, 1 min), and a final 7-min (or 30-min for DGGE; [33]) extension at 72°C. Successful PCR amplification was verified by agarose gel electrophoresis, and gene amplicons were purified using the MinElute clean-up system (Qiagen, Hilden, Germany). PCR products were quantified using a NanoDrop ND-1000 UV-Vis Spectrophotometer (NanoDrop Technologies, Wilmington, DE, USA).

### Molecular Fingerprinting of Fungal Communities

For DGGE, PCR amplicons were digested with Mung Bean Nuclease for 15 min at 37°C to remove single-stranded residues. A total volume of 12 µl contained 1 × Mung Bean buffer (Promega, Alexandria, NSW, Australia), 0.1 U Mung Bean Nuclease (Promega), and 300 ng of purified PCR product. Digests were spiked with 3 µl of dye (0.05% [wt/vol] xylene cyanol, 70% glycerol, in water, pH 8.0) and loaded onto a 6% [wt/vol] polyacrylamide gel (acrylamide plus *N*,*N*’-methylenebisacrylamide [37.5∶1; wt/wt]). An optimal separation was achieved by a gradient of 15–35% (vol/vol) denaturants (100% denaturant was defined as 7 M urea and 40% [vol/vol] formamide). Selected PCR samples and Marker V (Nippongene, Tokyo, Japan) were loaded onto all gels and served as references. DGGE was performed with the Ingeny PhorU System (Ingeny, Goes, The Netherlands) in 1 × TAE buffer (40 mM Tris, 20 mM acetic acid, 1 mM EDTA, pH 8 with NaOH) at 60°C for 18 h at 50 V. Gels were rinsed in water, stained for 30 min in 10,000 times diluted SybrGold nucleic acid stain (Invitrogen), destained for at least 2 h in double distilled water, and photographed under UV transillumination.

### Construction of Clone Libraries from Selected Rumen Samples

Fungal ITS1 regions were cloned from selected amplified DNA samples using the TA Cloning Kit (Invitrogen). Randomly selected clones were subjected to vector-targeted PCR with the primers Gem2987F (5′-CCCAGTCACGACGTTGTAAAACG-3′) and Top168R (5′-ATGTTGTGTGGAATTGTGAGCGG-3′). The resulting PCR products were purified, quantified, and sequenced at Macrogen Inc. (Seoul, Republic of Korea). In total, 401 sequences were obtained from 11 different clone libraries and deposited with GenBank (for accession numbers see Table 2). In addition, we deposited 16 sequences retrieved from the excision of DGGE bands (GenBank accession numbers JF423627-JF423642), and two clone sequences of each of three different isolates obtained from New Zealand ruminants in an earlier study (GenBank accession numbers JF423621-JF423626; [10]). Isolates *Piromyces communis* NZB19 and *Caecomyces* sp. NZB7 were originally isolated from grazing bulls and isolate *Neocallimastix frontalis* PNK2 from a grazing sheep in New Zealand and revived from the AgResearch culture collection.

**Table 2:** Clone library prefixes, numbers of clones sequenced from each library, and corresponding GenBank accession numbers.

Clone library prefix	Number of clones sequenced	GenBank accession numbers
S4-SG-ITS400Rw	48	JF423471-JF423518
S4-WG-ITS400Rw	51	JF423570-JF423620
S4-SI-ITS400Rw	51	JF423519-JF423569
C5-SG-ITS400Rw	36	JF423680-JF423706, JF423725-JF423733
C5-WG-ITS400Rw	25	JF423707-JF423724, JF423734-JF423740
C5-SI-ITS400Rw	25	JF423868-JF423892
D4-SG-ITS400Rw	34	JF423741-JF423774
D4-SI-ITS400Rw	30	JF423775-JF423804
D2-WG-ITS400Rw	38	JF423643-JF423679, JF423843
D3-WG-ITS400Rw	38	JF423805-JF423842
D1-SI-ITS400Rw	25	JF423844-JF423867 and JF423893

### Phylogenetic Analysis of Anaerobic Fungal ITS1 Sequences

To determine the phylogenetic affiliations of cloned ITS1 sequences, minimum free energy secondary structure information was obtained for all sequences (417 clone sequences obtained in this study and 380 reference sequences from isolates and environmental clone sequences deposited in the NCBI database) via the Vienna RNA secondary structure server [34]. The software 4SALE v1.6 was used to simultaneously align sequence and secondary structure information in an automated and impartial manner [35]. In addition to primary sequence information, 4SALE uses secondary structure information for sequence alignment. Because no consensus secondary structure exists for the ITS1 of the anaerobic rumen fungi, minor base changes and sequencing errors can cause major errors in the alignment. The alignment was therefore checked manually. If the secondary structure was very different to those of close relatives with the primary structure being highly similar, which is not expected biologically, the secondary structure and hence alignment of this outlier was adapted to the consensus, but the primary sequence was not changed. The resulting sequence alignment consisted of a total of 590 characters, 81 of which were conserved in 90% of the sequences. The alignment was imported into MEGA5 [36] and ARB [32] for subsequent tree construction and establishment of the taxonomic framework. For this, positions 105 to 372 were used (numbering after Tuckwell *et al.* [25]).

A total of 38 sequences were either too short, potentially chimeric or did not cluster reproducibly, and were therefore excluded from the alignment. These sequences were, however, included in the anaerobic fungal database we provide. They were incorporated into both the sequence and taxonomy files and referenced according to their accession numbers. Should these sequences turn out to represent the closest BLAST hits to a large number of pyrosequencing reads in future data sets and thus become of interest in future environmental studies, then further effort will have to be made to place these sequence types in the taxonomic scheme.

Phylogenetic trees were calculated in MEGA5 using the Neighbor-Joining and Unweighted Pair Group Method with Arithmetic Mean (UPGMA) algorithms, each with either Jukes-Cantor correction [37] or the Kimura 2-parameter distance model [38]. Trees were constructed using pairwise deletion and 1,000 replicates for bootstrapping analysis.

In addition to using matrix-based substitution models for tree construction, a consensus tree was calculated from 100 replicates using the character-based parsimony method (ordinary DNAPARS) in Phylip v3.69 [39]. Parameters were set to count all steps (sites unweighted).

Furthermore, a sequence alignment was created without the use of secondary structure information, fully reliant on manual arrangement of conserved and variable sequence motifs. From this alignment, a tree was constructed in MEGA5 using the Neighbor-Joining algorithm with Jukes-Cantor correction and pairwise deletion. One thousand replicates were used for bootstrapping analysis.

Finally, the topologies of all six trees were compared and a pragmatic taxonomic naming scheme was established.

### Cloning of Excised DGGE Bands

Individual bands in DGGE gels were excised from the gels and washed in 50 µl of molecular biology grade water before they were soaked in 30 µl of water overnight at 4°C to elute the DNA. DNA was then re-amplified using the DGGE primer pair ITS1F/ITS400R without the GC-clamp as described above. Successful PCR amplification was verified by agarose gel electrophoresis and purified using the MinElute Purification kit (Qiagen). Purified PCR products were quantified by NanoDrop, ligated with the TA Cloning Kit, and transformed into chemically competent *E. coli*. DNA was extracted from the resulting transformants and amplified using the primer pair ITS1F/ITS400R-GC. These products were checked by DGGE to show that they migrated to the expected position before they were subjected to vector-targeted PCR as described above and sequenced at Macrogen Inc.

### Construction of Pyrosequencing Libraries

Six of the rumen samples analyzed here by means of DGGE and clone libraries were amongst other samples selected for 454 Titanium pyrosequencing of fungal ITS1 genes and are part of a separate study (S. Kittelmann, H. Seedorf, W. A. Walters, J. C. Clemente, R. Knight, J. I. Gordon, and P. H. Janssen, manuscript in preparation). DNA was extracted twice from one of these samples. The sequencing data produced for these seven DNA samples were used for validation of the anaerobic fungal database established in this study. Primers used for barcoded PCR amplification of anaerobic fungal ITS1 genes were synthesized by Integrated DNA Technologies Inc. (Coralville, IA, USA; Table 1). Primers contained the adaptors A (5′-CCATCTCATCCCTGCGTGTCTCCGACTCAG-3′) or B (5′-CCTATCCCCTGTGTGCCTTGGCAGTCTCAG-3′) for Titanium sequencing (454 Life Science, Branford, CT, USA), and a unique 12-base error-correcting Golay barcode was attached to adaptor A for sample identification [40]. Each PCR contained 36 µl of 5 PRIME HotMasterMix (5 PRIME Inc., Gaithersburg, MD, USA), 32 µl of 0.5 µM non-barcoded primer and 8 µl of barcoded primer (2 µM working concentration). Before the addition of template DNA, an aliquot of 19 µl was transferred into a sterile tube and served as a no-template negative control. Three microliters of DNA (from a stock at approximately 40 ng µl^–1^) were added to the remaining 57 µl, and this then divided into 3 aliquots of 20 µl each. Amplification was performed on a PTC-225 PCR cycler (Bio-Rad, Hercules, CA, USA), with initial denaturation at 95°C for 2 min, 35 cycles denaturing (95°C, 20 s), annealing (50°C, 20 s) and elongation (65°C, 1 min), and a final 7-min extension at 65°C. Triplicates were pooled, and correct sizes of PCR products and signal absence from the negative controls were verified by agarose gel electrophoresis. PCR products were quantified using the Quant-iT dsDNA BR assay kit (Invitrogen), normalized and pooled. A total of 1 µg of DNA was loaded onto a 1% agarose gel (wt/vol). The band was visualized, excised under blue light transillumination, and subsequently gel purified using the Qiaquick gel extraction kit (Qiagen). The gel-purified amplicon pool was quantified in triplicate with the Quant-iT dsDNA HS assay kit (Invitrogen), diluted to obtain a concentration of 2×10^5^ copies ul^–1^, and subject to emulsion PCR. DNA-positive beads were enriched, counted on a Z1 particle counter (Beckman Coulter, Brea, CA, USA), and loaded onto a picotitre plate for pyrosequencing on a Genome Sequencer FLX machine (454 Life Sciences). Only sequences >200 bp in length and with average quality scores >25 were included in the analysis. Sequence reads were assigned to corresponding rumen samples by examining the 12-bp error-correcting Golay barcodes. Sequence data were phylogenetically assigned by BLAST using the QIIME pipeline (v1.2.1; [41]) and the sequence and taxonomy files developed in this study (available from the authors upon request). Pyrosequencing reads used in this study will be deposited in MG-RAST [42] as part of a larger dataset (S. Kittelmann, H. Seedorf, W. A. Walters, J. C. Clemente, R. Knight, J. I. Gordon, and P. H. Janssen, manuscript in preparation).

### Statistical Analyses

qPCR data were analyzed using the Rotorgene 6000 series software version 1.7 (Corbett Life Science) and subsequently exported to Excel (Microsoft Corp., Redmond, WA, USA) for further evaluation. DGGE banding patterns were analyzed with Bionumerics software v4.0 (Applied Maths Inc., Sint-Martens-Latem, Belgium). Cluster analysis was performed using UPGMA with Pearson correlation. To test for differences between treatment groups, normalized band intensities were exported from Bionumerics, and the differences between treatments assessed by permutational multivariate analysis of variance using the software package R (PERMANOVA; [43]).

Simpson’s dominance index was used as a measure of community diversity, with 1 indicating maximum diversity. This was calculated for each clone library according to Simpson [44] using the software PAST [45]. For pairwise comparisons between different communities, we used PAST to calculate the Morisita index of community similarity, which takes into account both species diversity and abundance [46]. Morisita indices range from 0 to 1, with 1 indicating that the two communities analyzed are identical.

Data from pyrosequencing and clone libraries were subjected to UPGMA cluster analysis using the Bray-Curtis dissimilarity distance metric in QIIME [47].

## Results and Discussion

### Primer Design and Validation

Several primers are described in the literature for the amplification of fungal ITS1 sequences. Three of these are universal and also amplify DNA from plants and fungal endophytes (Table 1). There are only a limited number of primer combinations for targeted amplification of the ITS1 from anaerobic fungi from environmental samples (Table 1). Not all of the specific primers are guaranteed to pick up the full diversity of fungal ITS1 genes. Edwards *et al.* [23] compared the primer MN100F (a modified version of primer AF100R previously used for quantitative PCR [48]) to sequences obtained from an axenic culture of *Anaeromyces* sp. GE09 and other members of the *Neocallimastigomycetes*, and concluded that the target region of the primer is not conserved. Our *in silico* analysis of primer binding to 351 anaerobic fungal sequences from isolated species and clones revealed that only 78.6% of all sequences (with sequence information in the primer binding region) had no mismatch to the primer MN100F, which is routinely used in combination with universal primer MNGM2R (84.2% of sequences with no mismatch). We therefore used the universal forward primer ITS1F instead (84.7% of sequences with no mismatch; [49]) and designed a new specific reverse primer, named ITS400Rw, with a binding site within the 5.8S region, downstream of variable region IV of the ITS1 gene but upstream of the binding site of universal primer MNGM2R (Table 1). This primer targets 86.3% of sequences. A modified version, ITS400R, without the degeneracy (Table 1), was used for DGGE fingerprinting. Both primers, ITS400R and ITS400Rw, in combination with ITS1F, allow amplification of full ITS1 sequence information (variable region I to variable region IV) for DGGE profiling and clone library generation, and provide an improved coverage of the total anaerobic fungal sequence diversity. For qPCR, we used the published primer pair AF1482F and AF100R [48]. Specificity of the three primer combinations was confirmed by cloning and sequencing amplicons generated from DNA extracted from a rumen sample collected from sheep S4 fed on summer pasture. All of the 138 cloned amplicons that were sequenced were from members of the *Neocallimastigomycetes*, confirming the specificity of the primer pairs (data not shown). These primer pairs were subsequently used to analyze fungal ITS1 regions in DNA extracted from a variety of rumen samples.

### Selection of Rumen Samples for Broadening Anaerobic Fungal Sequence Diversity

**Figure 1:**
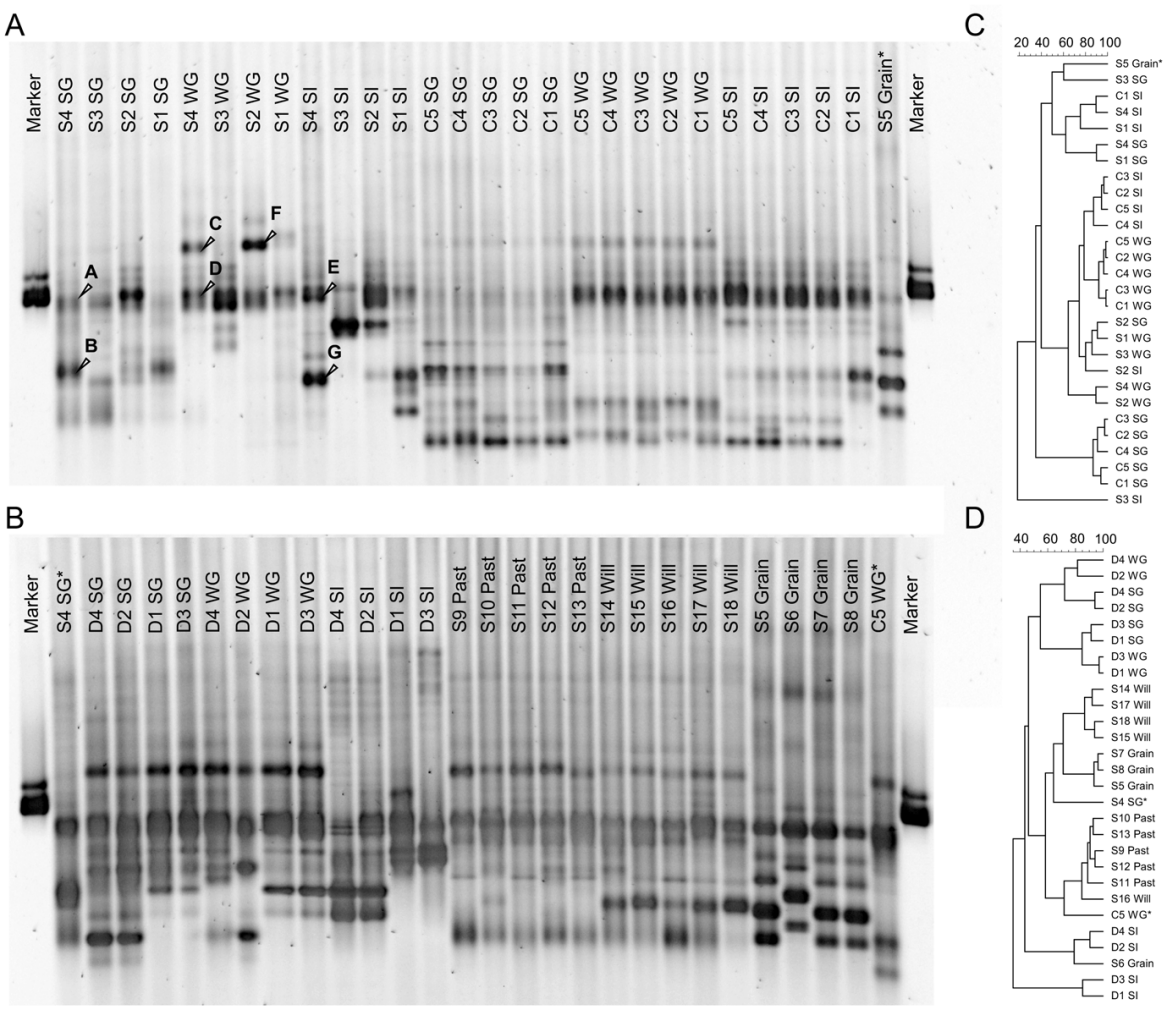
Diversity of anaerobic fungal ITS1 genes in rumen samples from sheep, deer, and cattle. DGGE profiles (panels A and B) and corresponding UPGMA cluster analyses (panels C and D, respectively) based on Pearson correlation of similarity between profiles of fungal ITS1 sequences amplified from fungal communities in rumen samples of (A, C) four sheep and five cows, and (B, D) four deer and 14 sheep feeding on summer pasture (SG), winter pasture (WG), silage (SI), autumn pasture (Past), willow (Will), or a concentrate-based diet (Grain). The scale bars above the dendrograms indicate the similarity between fungal community profiles in percent. Samples used as internal standards are marked with an asterisk.

We performed DGGE fingerprinting (Figure 1) and quantitative real-time PCR of anaerobic fungal ITS1 amplicons from 53 rumen samples to select a diverse range of samples, which were then used to generate more ITS1 sequences via clone libraries for an in-depth phylogenetic analysis of anaerobic fungi and the construction of a taxonomic framework applicable to high-throughput next generation sequencing data. Real-time PCR and DGGE fingerprinting furthermore allowed us to (a) gain insight into quantitative differences of anaerobic fungal communities (see Text S1), (b) assess the diversity and degree of animal-to-animal variation of fungal communities (see Text S2), and (c) evaluate the influence of ruminant host species and/or diet on fungal communities.

### Effect of Diet and Host Species on Rumen Fungal Communities

The same group of cattle and sheep were fed three different diets (summer pasture, winter pasture, and silage), and DGGE patterns indicated that there were diet and ruminant species effects on the fungal communities (Figure 1A, B). PERMANOVA tests suggested that the fungal community varied by ruminant species (*P* = 1×10^−4^) and by diet (*P* = 1×10^−4^), and that there was an interaction of ruminant species and diet (P = 1×10^−3^). DGGE profiles of the fungal communities of red deer clustered into two pairs (animals D1/D3 and animals D2/D4) on each of the three diets (summer pasture, winter pasture, and silage; Figure 1C, D). Apparently, in this experimental group of red deer so far unidentified animal-related factors outweighed the effect of the administered diets. However, there was evidence for a diet effect on fungal communities in red deer (*P* = 0.029). The additional groups of sheep also had different fungal communities, by diet (*P* = 1×10^−4^), although in this case these were different individuals on each diet. Still, it showed that each group had a distinct fungal community. These results suggests that anaerobic fungal communities do not randomly assemble in the rumen, but that different species occupy distinct environmental niches influenced by diet and/or by the host animal.

### Collection of ITS1 Gene Sequence Data

To date, the taxonomic assignment of anaerobic fungi has been largely hampered for two major reasons. Firstly, there is no stable and extendable phylogenetic framework containing type species of all characterized genera linked to validated and curated gene sequence data. Secondly, sequences from environmental clones or isolates are deposited in publicly-available databases under controversial taxonomic descriptions [26]. Taxonomic ranks associated with sequences deposited in GenBank (NCBI; [50]) are frequently used as references for BLAST-based taxonomic assignment of reads generated by next-generation-sequencing technologies, e.g., using the software QIIME [41]. To extract maximal value from large datasets, a thorough phylogeny-based taxonomic guide is needed that is extendable as new groups are discovered and more sequences of type species and strains become available. In the absence of a trustworthy formal taxonomic scheme that is based on valid descriptions of Linnaean taxa linked to curated gene sequence data, a more pragmatic approach seems warranted. We derived a taxonomic identification guide from a consensus tree that was calculated based on curated anaerobic fungal ITS1 gene sequence data, and applied a nomenclatural scheme that employs known genus and species designations where possible, and uses temporary designations at approximately genus and species rank where the formal taxonomy is still unresolved.

On 11 June 2011, NCBI’s GenBank database contained 191 ITS1 gene sequences belonging to isolated and cultivated anaerobic fungi [50]. We significantly increased the diversity of anaerobic fungal sequences by constructing clone libraries from those rumen samples that generated distinctly different DGGE profiles (Figure 1). Using the primer pair ITS1F and ITS400Rw, we amplified and sequenced 401 ITS1 genes from DNA extracted from 11 samples of rumen contents from 5 different animals on a variety of diets (Table 3). A manual alignment was created from 197 sequences of isolates (6 additional sequences were obtained in this study), 183 representative environmental sequences of recently detected novel groups of anaerobic fungi [21], [22], [26], [28], and 417 clone sequences (401 sequences from the libraries, 16 sequences from excised DGGE bands; this study). Clustering of sequences based on a manual alignment of the primary structure information alone was compared to the clustering based on automatic sequence and secondary structure alignment using the software 4SALE [35]. Sequences of 38 potential isolates, available in GenBank, were not used for tree construction because they were either too short, potentially chimeric, or showed hardly any consensus with the other sequences even in conserved regions. These sequences were, however, incorporated into the reference database we produced (comprising of a sequence and taxonomy file). In the first version of our reference database, their taxonomic ranks are only identified based on their corresponding accession numbers. Future studies on anaerobic fungal community structure will have to answer whether these sequences are indeed genuine and abundant. If further related sequences should appear, then efforts should be made to incorporate those into a tree-based phylogeny and to name the new clusters.

**Table 3:** Relative abundances of major phylogenetic groups of anaerobic fungi obtained from clone libraries.

Cluster^a^	Contribution to libraries (%)											Assignment of sequences from bands in DGGE gels
	Samples from sheep			Samples from cows			Samples from deer					
	S4 SG	S4 WG	S4 SI	C5 SG	C5 WG	C5 SI	D1 SI	D2 WG	D3 WG	D4 SG	D4 SI	
	(48)^b^	(51)	(51)	(36)	(25)	(25)	(25)	(38)	(38)	(34)	(30)	
Caecomyces 1				25.0	4.0					23.5		
Neocallimastix 1	8.3	37.3	11.8		24.0	56.0	84.0	52.6	34.2	8.8		A, D^c^
Orpinomyces 1				36.1	12.0	8.0						
Orpinomyces 4			19.6		4.0							E
Orpinomyces 5				27.8								
Orpinomyces 6				5.6	16.0			7.9		35.3		
Piromyces 1			27.5									G
Piromyces 2	22.9	15.7			16.0			10.5				
Piromyces 3	12.5		23.5			12.0						
Piromyces 4									5.3		100	
Piromyces 5									39.5	2.9		
Piromyces 6			13.7			8.0						
Piromyces 7	12.5	5.9			12.0							
AL6									2.6			
BlackRhino			2.0			16.0	16.0					
SK1	2.1	41.2			8.0			5.3	13.2			C, F
SK2								5.3	5.3			
SK3	8.3			5.6	4.0			18.4		29.4		
SK4	33.3											B
Unassigned			2.0									

**Figure 2:**
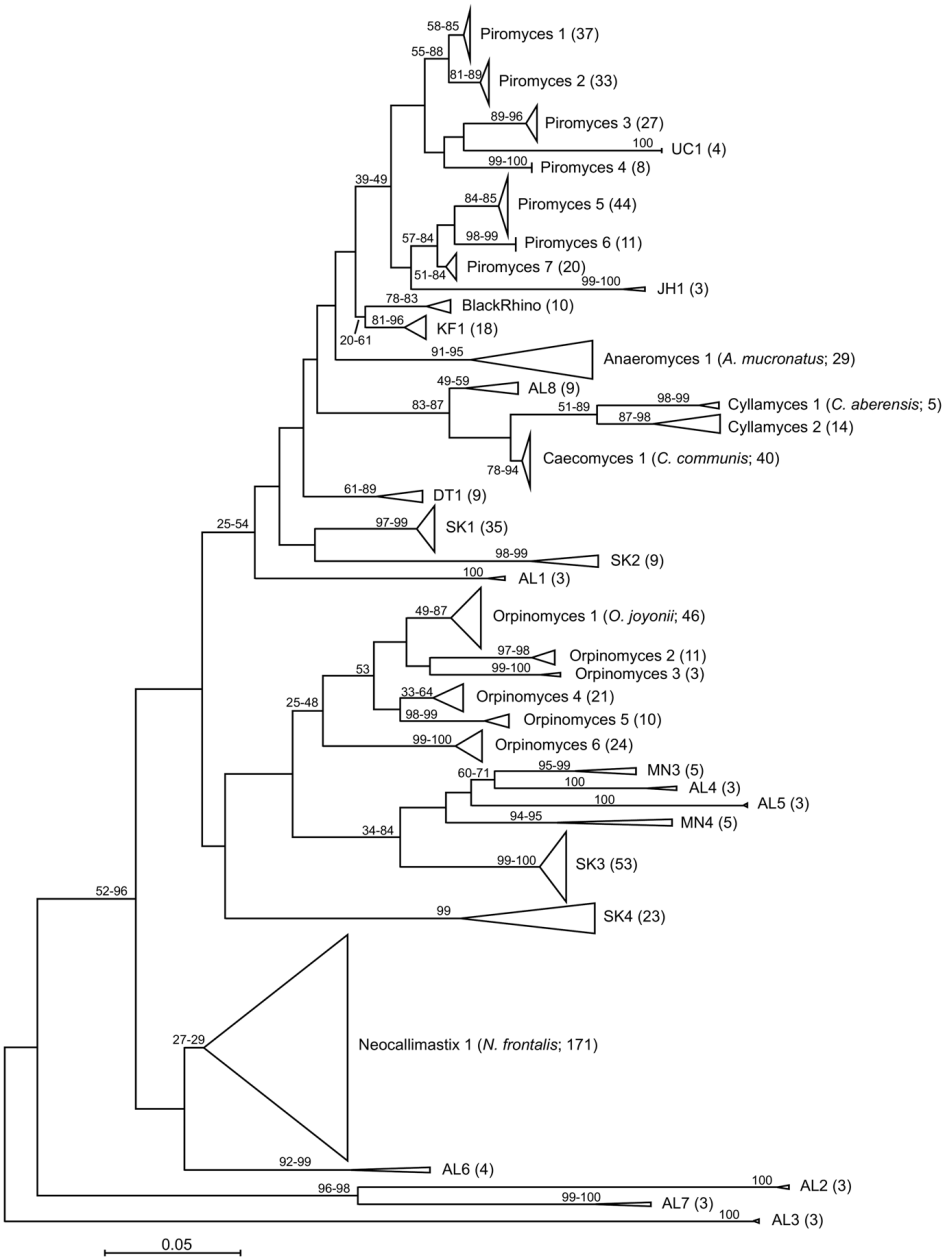
Proposed working taxonomy of the anaerobic fungi based on ITS1 genes. Phylogenetic tree constructed from 759 fungal ITS1 gene sequences (positions 105 to 372; numbering after Tuckwell *et al.* [25]) using the Neighbor-Joining (NJ) algorithm with pairwise deletion and Jukes-Cantor (JC) correction [37]. In total, four different trees were calculated in MEGA5 with two different treeing algorithms and two different correction models (NJ-JC, NJ-Kimura, UPGMA-JC, and UPGMA-Kimura), and values on branches indicate the range of bootstraps obtained across the 4 trees (from a total of 1,000 replicates for each tree). Bootstraps are not shown if branching was unstable or values <20. The scale bar indicates 0.05 nucleotide substitutions per nucleotide position. For clarity, coherent groups are collapsed into triangles, with the number of sequences shown in parentheses after the group designation and the corresponding type species (if available). Assignment of individual sequences to the corresponding clusters can be deduced from the taxonomy file available from the authors upon request. The following cluster designations differ from previous naming schemes (former name and literature reference are given in brackets): AL1-8 (NG1-8; [21]), MN3-4 (NG3-4; [22]), Cyllamyces 1–2 (NG1 *Cyllamyces*; [22]), Orpinomyces 1–6 (*Orpinomyces*/*Piromyces* II; [25]), Piromyces 1–7 (*Piromyces* III; [25]).

Clone libraries were constructed from samples from sheep S4, cow C5 and deer D1, D2, D3, and D4 fed summer pasture (SG), winter pasture (WG), or a silage-based diet (SI). Summarized abundances of major genera and novel groups across all analyzed samples are given as well as the taxonomic assignments of sequences from bands in DGGE gels.

a The cluster designations are those used in Figure 2.

b In parentheses are the total number of sequences in each library.

c Letters indicate the clusters containing sequences from bands marked by the same letters in DGGE gels shown in Figure 1.

### Revised Taxonomy of Anaerobic Fungi and Detection of Novel Groups

Phylogenetic analysis of the highly variable, short ITS1 sequences resulted in groupings with a generally relatively low bootstrap support. However, all six treeing analyses that were performed (see Materials and Methods section; Figure 2) recovered the same groupings and allowed the ITS1 sequences to be grouped into 37 reproducible clusters. All sequences except one grouped reproducibly within the same clusters using all six methods, and the inconsistently-grouping sequence (GenBank accession number HQ263338) formed an adjacent sister group only when Maximum Parsimony was used for tree construction. To produce a taxonomic scheme compatible with large pyrosequencing data sets, we gave those stable clusters working designations. Each of the 37 clusters contained at least 3 sequences. Eighteen clusters contained at least one reference sequence from an anaerobic fungal isolate (Piromyces 1, 2, 5 and 7, UC1, Anaeromyces 1, AL8 [corresponding to NG8 of Liggenstoffer *et al.* [21]], Cyllamyces 1 and 2, Caecomyces 1, DT1, SK2 and SK3, Orpinomyces 1, 2 and 4, MN4 [corresponding to NG4 of Nicholson *et al.* [22]], and Neocallimastix 1). Isolates that grouped within SK2 and SK3 were previously described as *Anaeromyces* spp. However, since these clusters were clearly separated from the true *Anaeromyces* spp., and potentially represent novel species or genera, we have designated these clusters as SK2 and SK3. Seventeen clusters contained only environmental clone sequences, and with the exception of JH1 [28] all remaining clusters contained sequences from at least two different studies. These were Piromyces 3 and 6, BlackRhino (BlackRhino04IGVMQ, GQ738584; [21]), KF1 [26], SK1 and SK4 (this study), AL1 to AL7 (corresponding to NG1 to NG7 of Liggenstoffer *et al.* [21]), Orpinomyces 3 and 6, and MN3 (corresponding to NG3 of Nicholson *et al.* [22]). Two further clusters, Piromyces 4 and Orpinomyces 5, contained only sequences obtained from New Zealand ruminants in this study. By adding our new sequences from domesticated ruminants, we expanded the diversity within 10 groups containing isolates (Piromyces 1, 2, 5 and 7, Caecomyces 1, SK2 and SK3, Orpinomyces 1 and 4, and Neocallimastix 1) and detected two novel clusters within the genera *Piromyces* (cluster Piromyces 4) and *Orpinomyces* (cluster Orpinomyces 5). Obviously, equating these new clusters with new species is still speculative at this stage. Several of our sequences grouped with the BlackRhino cluster (a sequence group first obtained from a rhinoceros [21]). One single sequence from our study clustered into AL6.

We further detected novel sequence types in our dataset that formed the new clusters SK1 and SK4 which did not group with previously-named genera. The environmental sequences from earlier studies that also grouped within these novel clusters had previously not been classified or had been wrongly assigned to one of the six known genera even though they are clearly different. The novel clades, referred to as SK1, SK2, SK3, and SK4 (Figure 2), comprised 23.4% of all sequences retrieved in our libraries (Table 3). Sequences clustering in novel cluster SK1 were so far almost exclusively found in DNA extracted from rumen samples of New Zealand ruminants. However, a single environmental sequence obtained from the dung of a bison at Wind Cave National Park, SD, USA [28] also fell into the novel SK1 cluster. In addition to sequences from this study, cluster SK2 contained five clone sequences from isolate “*Anaeromyces*” GE09. Sequences belonging to novel cluster SK3 were closely related to the sequence of isolate “*Anaeromyces*” GA-01-CIRG (GenBank accession FJ889133; sequence similarity ≥99.0%) and also contained several environmental sequences from other studies [26], [28]. However, clusters SK2 and SK3 grouped away from true *Anaeromyces* spp. (Figure 2). Members of novel group SK4 showed highest sequence similarity to environmental sequence S131 (GenBank accession AM690064 [25]; sequence similarity 91.4–100%).

ITS1 sequences in GenBank that are assigned to the genus *Piromyces* do not group monophyletically [17], [25]. Since sequence information for the type species *Piromyces communis* [51] is not available, the genus name *Piromyces* cannot be easily allocated to any of the sequence clusters. The sequence of *Piromyces* sp. PrI (GenBank accession AY429665), the sole sequence representative of the genus *Piromyces* used by the Fungal Barcoding Consortium [16], was used as a reference sequence to define Piromyces cluster 1. The large majority of sequences from other “*Piromyces*” isolates clustered within the group designated as *Piromyces* III by Tuckwell *et al.* [25] and fell into clusters that grouped with our Piromyces 1. We therefore decided to designate the seven sequence clusters (including Piromyces 1) that grouped together with all of the treeing methods used as provisionally “true” members of *Piromyces* (Figure 2), named Piromyces 1 to Piromyces 7. Clusters UC1 and JH1 grouped within this *Piromyces* assemblage using the Neighbor-Joining algorithm, but outside when UPGMA clustering was used. Therefore, these two sequence clusters are not designated as members of *Piromyces*, but referred to by their trivial cluster names. Further study may reveal their actual relationship to members of the genus *Piromyces*.

We found only one cluster that we regarded as true *Anaeromyces*, which was based on the presence of the validated type species *Anaeromyces mucronatus* YE505 (sequence information was kindly provided by Tim McAllister, Agriculture and Agri-Food Canada; [14]). Other sequences that have previously been labeled as *Anaeromyces* are now designated as members of clusters DT1, SK2, and SK3.

The phylogenetic analysis resulted in 2 coherent clusters that we designated *Cyllamyces* (Figure 2). Cyllamyces 1 contains the validated type species *Cyllamyces aberensis* E014 and E017 (AY997042 and FJ483845; [52]). Cyllamyces 2 consists of a variety of *Cyllamyces* isolates described by several different published studies and the sequence of the potentially mis-named isolate *Caecomyces sympodialis* W101.

Phylogenetic placement of reported members of the genus *Caecomyces* is a rather complicated task, as outlined previously [26]. Neither ITS1 sequence information nor a live culture is available for *C. equi*, the type species of this genus, but Ho and Barr [53] propose that *C. equi* and *C. communis* may in fact represent the same species. To define the genus *Caecomyces*, we used as reference sequences only those sequences that are supported by morphological data, which are *Caecomyces communis* CY50 (DQ067605; [54]), *Caecomyces sympodialis* W101 (DQ067604; [54]), *Caecomyces* sp. CR4 (AB334759; [55]), and two sequences from the New Zealand isolate *Caecomyces* sp. NZB7 (JF423621 and JF423622; [10]). The so-far unpublished sequence of isolate NZB7 has previously been used to produce sequence fingerprints for the ITS1 variable regions for the genus *Caecomyces* [25]. We designated the coherent cluster containing these and several sequences from isolates closely related to *Caecomyces communis* CY50 as the true *Caecomyces* cluster. *Caecomyces sympodialis* W101, however, was more closely related to true *Cyllamyces* spp. and was therefore incorporated into the Cyllamyces 2 cluster (Figure 2), as described above. The cluster containing the sequence of the isolate *Caecomyes* sp. CR4 together with environmental sequences belonging to clusters NG8 of Liggenstoffer *et al.* [21] and NG2 of Nicholson *et al.* [22] grouped slightly outside of these three clusters. We decided not to use the names NG1 to NG4 of Nicholson *et al.* [22] and NG1 to NG8 of Liggenstoffer *et al.* [21] because the same designations in these studies refer to different groups. We therefore use a new series of designations, representing the initials of the first author to describe a sequence in the corresponding cluster. Due to the larger sequence depth within group NG8 of Liggenstoffer *et al*. [21] compared to the corresponding group NG2 of Nicholson *et al*. [22], we decided to adopt the designation AL8 for the wider group (Figure 2). The “super-cluster” containing AL8, *Cyllamyces* and *Caecomyces* was coherent using all six treeing methods.

Despite the lack of sequence information for the type species of the genus *Orpinomyces*, *O. bovis* [56], this genus is represented by numerous sequences of isolated species that clustered coherently, and these were used to define the genus (Figure 2). We have identified 6 subgroups, labeled Orpinomyces 1 to Orpinomyces 6.

The cluster designated as the genus *Neocallimastix* contains sequences from the validated type species *Neocallimastix frontalis* (AY429664 and AF170202; [16], [57]), but shows great phylogenetic depth. It could not easily be further divided into different subclusters. This was not what we intended, because we wanted to avoid large undifferentiated groups. However, we could not subdivide this group based on the available sequence information, and so, for now, are forced to leave it as a probable genus with one working “species”, realizing that the depth is indicative of more than one true species. This would mask shifts within the genus when next-generation sequencing data are analyzed.

Our assignment and designations differ from those suggested in earlier studies. Comparing our revised taxonomy to the taxonomic assignment of sequences deposited in the GenBank database, we believe that >29% of ITS1 sequences derived from anaerobic fungal isolates or clones are misnamed at the genus level. This highlights the need for detailed guidelines for the morphological identification of anaerobic fungi, and for correctly identified pure cultures with corresponding sequence data. Furthermore, database curation and proper phylogenetic placement of sequences is essential in order to avoid erroneous assignment of hundreds or thousands of reads from high-throughput next-generation-sequencing data sets. The phylogenetic framework constructed and the taxonomy suggested here offer starting points for re-defining the known anaerobic fungal genera based on their ITS1 gene sequences and allow for future inclusion of potentially novel genera or species. The taxonomy and sequence file derived from our phylogenetic approach are available from the authors upon request via email.

### Comparison of Fungal Community Structure Assessed from Clone Libraries and Pyrosequencing Using the Revised Anaerobic Fungal Taxonomy

**Figure 3:**
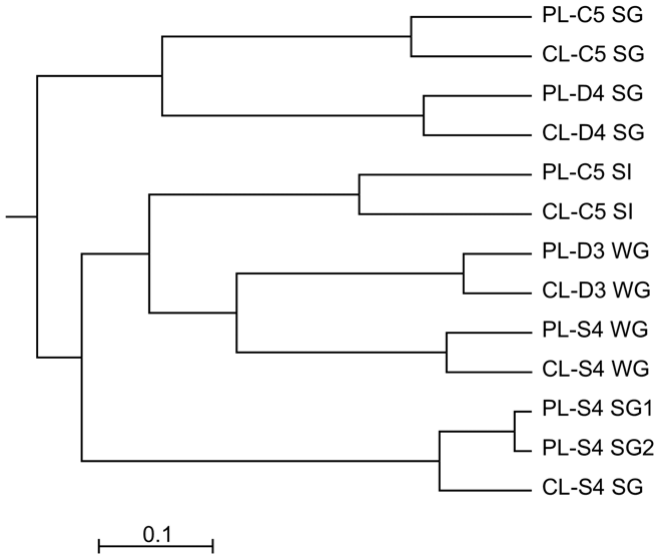
Comparison of sequence libraries generated by cloning and by barcoded pyrosequencing of ITS1 genes. Cluster analysis of clone libraries (prefixed CL) and pyrosequencing libraries (prefixed PL) constructed from 6 different rumen samples based on anaerobic fungal ITS1 gene sequences using the Bray-Curtis distance metric and UPGMA treeing. DNA was extracted twice from the rumen sample of sheep S4 on summer pasture, and two independent pyrosequencing libraries (suffixed 1 and 2) were constructed for this sample. Abbreviations are used as described in Figure 1. The length of the scale bar represents anaerobic fungal community dissimilarity of 10%.

Pyrosequencing data of anaerobic fungal ITS1 genes were generated from six rumen samples and successfully assigned to phylogenetic groups using the newly established taxonomic framework and curated sequence file. DNA was extracted twice from one of the samples, and the two lots of DNA treated as two separate samples, to give seven in total. The anaerobic fungal community composition in the pyrosequencing libraries was compared to that in parallel traditional clone libraries by performing UPGMA cluster analysis (Figure 3). Pyrosequencing libraries were always most similar to the corresponding clone libraries. The average dissimilarity between anaerobic fungal community composition in clone libraries and the corresponding pyrosequencing libraries was 18.5% ± 6.0%. The communities in the replicated sample analyzed by pyrosequencing showed the smallest difference (2.9% dissimilarity). Several groups of anaerobic fungi were detected using pyrosequencing but were not obtained in the traditional clone libraries. This finding was expected because of the greater number of sequences generated using the newer technology. Using the taxonomic framework proposed here, a total of 99.5 ± 0.7% of all pyrosequencing reads could be assigned a taxonomic rank across the seven rumen samples that we analyzed.

### Diversity of Anaerobic Fungi in Selected Rumen Samples from New Zealand

Fingerprinting analysis by DGGE allowed us to select 11 different rumen samples for the construction of clone libraries and detailed phylogenetic analysis. According to Simpson’s index of diversity calculated from the presence and abundance of OTUs in the clone libraries, anaerobic fungal diversity was similar in the sheep (0.74 ± 0.07; mean ± standard deviation) and the cattle (0.72 ± 0.12), and lower in the deer (0.45 ± 0.31). The clone libraries showed a mean similarity of only 0.28 (Morisita Index of similarity; range: 0–0.9), confirming that samples harboring highly different anaerobic fungal communities had been selected for the construction of clone libraries. The phylotypes obtained in this study were affiliated with four known genera of anaerobic fungi, namely *Piromyces*, *Neocallimastix*, *Orpinomyces* and *Caecomyces*, and with clusters AL6, BlackRhino, and the four novel clusters SK1 to SK4 (Table 3).

*Piromyces* and *Neocallimastix* spp. were the most abundant anaerobic fungi in all clone libraries analyzed in this study, making up 32.7% and 26.4% of all sequences, respectively (Table 3). Representatives of these two genera were also prominent in samples collected from 19 ruminant animals by Liggenstoffer *et al.* [21], with *Piromyces* and *Neocallimastix* representing 28.7 and 15.1% of the total fungal communities in these samples, respectively. Isolated members of these two genera are known to possess high cellulolytic and xylanolytic activities [4], [58] and are apparently more effective at degrading stem fragments of ryegrass than *Caecomyces* spp. [10]. Clones belonging to the genus *Orpinomyces* contributed 15.0% of the total number of sequences analyzed here (Table 3). *Orpinomyces* spp. have so far been observed to be more abundant in the rumens of animals fed grain compared to high fiber diets [59]. However, Li *et al.* [60] showed that *Orpinomyces* sp. strain PC-2 possesses cellulases and xylanases that are structurally related to those of *Neocallimastix patriciarum*. Competition for substrate between these two different genera may explain the increased abundance of *Orpinomyces* spp. in animals with fewer *Neocallimastix* spp. (Table 3).

Sequences assigned to novel cluster SK1 were found in all three species of ruminants, but occurred almost exclusively when the animals were fed winter pasture. Sequences clustering within novel cluster SK2 were only retrieved from rumen samples of deer on a winter pasture diet. SK3-related sequences were obtained from sheep, deer, and cattle feeding on both summer and winter pasture, whereas SK4-related sequences were exclusively found in the sample of sheep S4 on summer pasture. No sequences representing the anaerobic fungal genera *Anaeromyces* and *Cyllamyces* were obtained from the rumen samples analyzed in this study, although polycentric species characterized as *Anaeromyces* were previously isolated from New Zealand ruminants (Naylor GE & Joblin KE, unpublished).

### Conclusions

Our findings indicate that the diversity of anaerobic fungi in the rumens of domesticated ruminants is greater than previously reported. Pre-screening of samples and the construction of clone libraries from those samples that were most diverse allowed a considerable expansion of the available sequence diversity of anaerobic fungi and the identification of four novel clusters that do not group with previously-named genera. Isolation efforts will have to be made to resolve their characteristics and functions in the rumen. DGGE fingerprinting revealed diet- or host-specific shifts in anaerobic fungal community structure. These findings indicate that fungal community composition in the rumen is not random. Anaerobic fungi should thus be included in the molecular monitoring of rumen microbial communities of animals showing different phenotypes (e.g., methane emissions, productivity). In future studies, the taxonomic framework proposed here will serve for simple but reliable BLAST-based sequence assignment of data obtained from high-throughput next-generation-sequencing techniques.

## Supporting Information

Text S1 Abundance and variability of anaerobic fungi.
(DOCX)

Text S2 Animal-to-animal variation of rumen fungal communities.
(DOCX)
