# Supplementary material for: A Proposed Taxonomy of Anaerobic Fungi (Class Neocallimastigomycetes) Suitable for Large-Scale Sequence-Based Community Structure Analysis
Source: PLoS One. 2012 May 16;7(5):e36866. doi: 10.1371/journal.pone.0036866 (PMC3353986; doi:10.1371/journal.pone.0036866)
Supplement: Text S1 — Abundance and variability of anaerobic fungi. (DOCX) [file pone.0036866.s001.docx]

**Text S1. Abundance and variability of anaerobic fungi**

We used qPCR to evaluate the abundance of anaerobic fungi in rumen samples from sheep (*n* = 4), cattle (*n* = 5), and red deer (*n* = 4) feeding on three different diets (summer pasture, winter pasture, or silage). We further analyzed three additional flocks of sheep. These had been feeding on a concentrate-based diet (*n* = 4), autumn pasture (*n* = 5) or willow (*n* = 5). Anaerobic fungi were universally present in all of these samples, but there was considerable variation between samples and no evidence of systematic differences. The abundance of fungal ITS1 gene copies in all 53 samples ranged from 5 × 10^-6^ to 0.22 copies per bacterial 16S rRNA gene copy (mean = 0.016).
