# Supplementary material for: A Proposed Taxonomy of Anaerobic Fungi (Class Neocallimastigomycetes) Suitable for Large-Scale Sequence-Based Community Structure Analysis
Source: PLoS One. 2012 May 16;7(5):e36866. doi: 10.1371/journal.pone.0036866 (PMC3353986; doi:10.1371/journal.pone.0036866)
Supplement: Text S2 — Animal-to-animal variation of rumen fungal communities. (DOCX) [file pone.0036866.s002.docx]

**Text S2. Animal-to-animal variation of rumen fungal communities**

DGGE revealed differences in ITS1 fingerprints obtained from the different samples (Fig. 1). In general, the number of bands was higher in cattle (8 to 10 clearly distinguishable bands) than in deer (6 to 8 bands) and sheep (2 to 8 bands). The degree of animal-to-animal variation was different in different groups of ruminants. While profiles of sheep from flocks 1 (animals S1 to S4) and 2 (animals S5 to S8) showed high animal-to-animal variation (mean similarities of 58.9% and 63.3%, respectively; Fig 1), sheep belonging to flocks 3 (animals S9 to S13) and 4 (animals S14 to S18) showed less variation between individuals fed on the same diet (mean similarity 86.5%; Fig. 1B). The profiles derived from red deer rumen samples D1 and D3 were highly similar on all three diets (mean similarity 91.2%), but showed only 47.3% similarity to the profiles of D2 and D4 (mean similarity 88.7% to each other; Fig 1B). In contrast to sheep (mean similarity 65.5%) and deer (mean similarity 61.5%), cattle displayed the smallest animal-to-animal variation. All five cows showed highly similar rumen fungal community profiles, independent of the feed (mean similarity 88.6%; Fig. 1A). Similar findings regarding animal-to-animal variation in these three groups of ruminants were reported for methanogenic archaea and ciliate protozoa [1,2]. Animal-to-animal variation of the rumen microbiota needs to be taken into account when planning animal trials, which aim at e.g. identifying methane inhibitors or selecting low-methane emitting animals. For example, we have previously found that ciliate communities can vary significantly between co-housed animals on the same diet [2], and that methanogen communities vary less than bacterial communities [1]. Knowledge of the degree of variation between equally treated animals optimizes experimental planning by maximizing the statistical power of experiments while at the same time minimizing the number of experimental animals [3].

DGGE bands were excised from the profiles generated from samples from sheep S4 on summer pasture, winter pasture, and silage, and sheep S2 on winter pasture, and DNA was cloned and sequenced. The sequences were compared to the parallel clone library of the corresponding sample for validation. Reasonable agreement between clone libraries and DGGE banding patterns was found and fungal groups that were represented by major bands were also prominent in the clone libraries of the corresponding samples (Fig. 1, Table 3). Cloning of DGGE bands proved difficult, however, and was not always successful, as cloned fragments from excised bands frequently migrated to different positions when re-run. DGGE was a useful screening tool to select samples that were different, but obtaining detailed phylogenetic information of the underlying community structure differences was not practical. We used the variation in DGGE banding patterns to select samples for the construction of clone libraries, and used the sequences from these libraries to expand the pool of anaerobic fungal ITS1 sequences for the establishment of a comprehensive phylogenetic framework.

1. Jeyanathan J, Kirs M, Ronimus RS, Hoskin SO, Janssen PH (2011) Methanogen community structure in the rumens of farmed sheep, cattle and red deer fed different diets. FEMS Microbiol Ecol 76: 311-326.

# 2. Kittelmann S, Janssen PH (2011) Characterization of rumen ciliate community composition in domestic sheep, deer, and cattle, feeding on varying diets, by means of PCR-DGGE and clone libraries. FEMS Microbiol Ecol 75: 468-481.

3. Thomas L (1997) Retrospective power analysis. Conserv Biol 11: 276-280.
